# *Cryptosporidium* Species Infections Detected from Fecal Samples of Animal and Human Hosts in South Africa: Systematic Review and Meta-Analysis

**DOI:** 10.3390/microorganisms12122426

**Published:** 2024-11-25

**Authors:** Mpho Tawana, ThankGod E. Onyiche, Tsepo Ramatla, Sebolelo Jane Nkhebenyane, Dennis J. Grab, Oriel Thekisoe

**Affiliations:** 1 Unit for Environmental Sciences and Management, North-West University, Potchefstroom 2531, South Africa; 2027028865@ufs4life.ac.za (M.T.); et.onyiche@unimaid.edu.ng (T.E.O.); oriel.thekisoe@nwu.ac.za (O.T.); 2 Department of Zoology and Entomology, University of the Free State, Phuthaditjhaba 9866, South Africa; 3 Department of Veterinary Parasitology and Entomology, University of Maiduguri, P.M.B. 1069, Maiduguri 600230, Nigeria; 4 Department of Life Sciences, Central University of Technology, Bloemfontein 9300, South Africa; snkheben@cut.ac.za; 5 Department of Pathology, Uniformed Services University of the Health Sciences, Bethesda, MD 20814, USA; dennis.grab@usuhs.edu; 6 Department of Tropical Medicine, Medical Microbiology and Pharmacology, University of Hawaii at Manoa, Honolulu, HI 96822, USA

**Keywords:** *Cryptosporidium* species, prevalence, South Africa

## Abstract

This study presents a systematic review and meta-analysis approach of *Cryptosporidium* species prevalence studies in animal and human hosts published between 1980 and 2020 in South Africa. Extensive searches were conducted on three electronic databases including PubMed, ScienceDirect and Google Scholar. The findings indicated an overall pooled prevalence estimate (PPE) of *Cryptosporidium* spp. infections in animals and humans at 21.5% and 18.1%, respectively. The PCR–RFLP appeared to be the most sensitive diagnostic method with a PPE of 77.8% for the detection of *Cryptosporidium* spp. infections followed by ELISA (66.7%); LAMP (45.4%); PCR (25.3%); qPCR (20.7%); microscopy (10.1%); IFAT (8.4%); and RDT (7.9%). In animal hosts, *C. parvum* had the highest PPE of 3.7%, followed by *C. andersoni* (1.5%), *C. ubiquitum* (1.4%) and *C. bovis* (1.0%), while in humans, *C. parvum* also had the highest PPE of 18.3% followed by *C. meleagridis* at 0.4%. The data generated in this study indicated that *Cryptosporidium* spp. infections were highly prevalent in both animals and humans in South Africa, especially in the KwaZulu-Natal and North West provinces. However, we further observed that there was a lack of prevalence studies for both animals and humans in some of the provinces. This study highlights the necessity for a “One Health” strategic approach promoting public hygiene, animal husbandry and regular screening for *Cryptosporidium* spp. infections in both animals and humans.

## 1. Introduction

South Africa is a developing country with over 59 million of human population and ranks number 13th in the global list for countries living in the poverty line [1,2]. Accounting for about 17% of the world’s HIV infections, South Africa’s population is already vulnerable to secondary opportunistic infections which include parasitic diseases such as cryptosporidiosis, giardiasis and toxoplasmosis [3]. Cryptosporidiosis is a zoonotic disease caused by protozoan parasites of the genus *Cryptosporidium.* Of the *Cryptosporidium* species causing disease in humans and animals, *C. hominis* and *C. parvum* are known to cause gastroenteritis among the general public [4,5]. While the disease is often self-limiting, cryptosporidiosis infection can be life-threatening to humans living with HIV/AIDS as well as children and young animals [6,7,8,9]. The main symptoms and signs for both animals and humans are watery diarrhea, weight loss, nausea, vomiting, fatigue and low-grade fever [10,11,12]. In southern Africa and Asia, about 2.9 million and 4.7 million cases of *Cryptosporidium* spp. infections have been reported among children less than 2 years old, respectively [13]. The first *Cryptosporidium* spp. infection cases to be recorded in South Africa were of four children in Durban in 1987, and since then, there has been an increase in the studies on *Cryptosporidium* parasitic prevalence in the country [14].

*Cryptosporidium* species can spread through the fecal–oral route either primarily (direct contact) or secondarily through the consumption of contaminated food or water with human or animal feces [15,16,17]. The annual quantity of excreted *Cryptosporidium* spp. oocysts by domestic animals globally has been estimated to be approximately 3.2 × 10^23^ [18]. Animals are important contributing factors of environmental contamination of *Cryptosporidium* oocyst distribution [19,20], spreading via water [21] or food [22]. *Cryptosporidium* spp. distribution and infection are exacerbated by their resistance to normal water treatment, including chlorination [23].

Diagnostic techniques for *Cryptosporidium* spp. infections includes microscopy, polymerase chain reaction (PCR) and enzyme-linked immunosorbent assay (ELISA) with reported sensitivities of 56.0% to 75.4%; 100%; and 89.7% to 100%, respectively [24].

Omolabi et al. [25] conducted a meta-analysis on *Cryptosporidium* species in humans from southern Africa. However, there is limited information on comprehensive data available to estimate the prevalence of *Cryptosporidium* spp. in humans and animals in South Africa. Hence, the premise of this review is founded around the wildlife–domestic–human interface, highlighting the shared, interconnected links between the health of humans, wildlife and domestic animals. Our meta-analytical approach allowed for the identification of study gaps, examination of the pooled prevalence for animal and human *Cryptosporidium* spp. in South Africa and further investigated the influence of risk factors such as age, sex, fecal consistency, HIV status and diagnostic techniques on the spread of the *Cryptosporidium* parasite.

## 2. Materials and Methods

### 2.1. Study Design

This systematic review and meta-analysis were conducted on published articles reporting *Cryptosporidium* spp. infections in South Africa, confirmed by examining feces of animals and humans for the presence of *Cryptosporidium* spp. oocysts using microscopy, immunological and molecular techniques.

### 2.2. Search Strategy and Criteria

Literature searches were conducted in PubMed, ScienceDirect and Google Scholar for articles published in English between 1980 and 2020 on the prevalence or epidemiology of *Cryptosporidium* spp. infections across South Africa in animals and humans. The search keywords were “prevalence”, “*Cryptosporidium*” and “South Africa”. Keywords used in the search were entered individually or in combination with the “AND” and/or “OR” operators. None of the authors of original studies were contacted for additional information and no attempt was made to retrieve unpublished articles. Titles and abstracts were scanned, and relevant full-text articles were downloaded and obtained through library resources and online platforms.

### 2.3. Inclusion and Exclusion Criteria

Articles were included only if they fulfilled the following inclusion criteria: cross section (prevalence study) conducted within South Africa, vertebrate host (humans or animals) used, study conducted on fecal samples, exact total numbers and positive cases clearly provided, sample size (≥25 for enabling statistical calculations) and written in English. Studies without these characteristics were all excluded such as review studies, studies on water, case report studies, ones with a lower sample size and ones not written in English.

### 2.4. Data Quality Control Measures

To confirm the methodological soundness of the research articles selected for quantitative synthesis, two authors independently used the Joanna Briggs Institute (JBI) Critical Appraisal Tools Checklist 2017 review guideline for prevalence studies. Studies that achieved a score of five or higher for the evaluation criteria were included.

### 2.5. Data Extraction

The data extraction protocol consisted of the name of the author and region, hosts, total sample size, number of positive cases, estimated prevalence, species of intestinal parasites, and diagnostic technique. Moreover, studies that were conducted in more than one province and those that had both animal and human studies simultaneously were separated accordingly.

### 2.6. Statistical/Meta-Analytic Procedures

The meta-analysis was performed using the Comprehensive Meta-Analysis (CMA) program [26]. The random effects model was used to estimate the pooled prevalence and corresponding 95% confidence interval (CI). Statistical heterogeneity between studies was estimated with Cochran’s *Q* statistic and I-square (*I*^2^) test (values of 25%, 50% and 75% were considered to represent low, medium and high heterogeneities, respectively). The funnel plot and Begg’s rank correlation test were used to evaluate the possibility of publication bias, where *p* < 0.05 was considered as indicative of statistically significant publication bias [27,28].

## 3. Results

### 3.1. Literature Search and Eligible Studies

**Figure 1:**
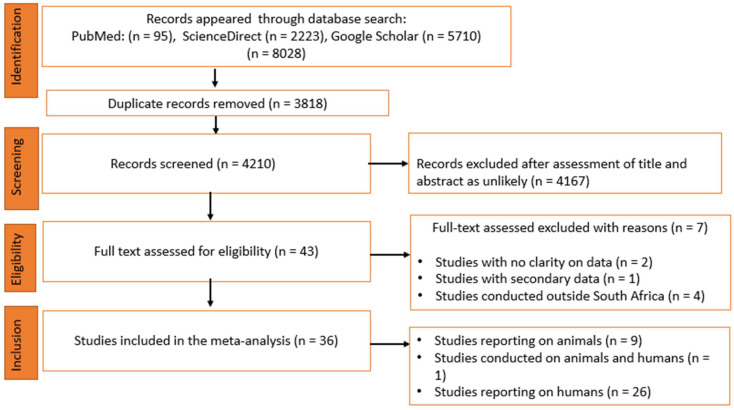
Flow chart of included studies according to PRISMA guidelines.

A total of 8028 studies were identified from three electronic databases, namely PubMed (95), ScienceDirect (2223) and Google Scholar (5710). After the removal of duplicates and a subsequent review of study titles and abstracts, 4167 studies were excluded and 43 studies were found to be eligible and subjected to full text evaluation for inclusion. Furthermore, seven studies were excluded for the following reasons: (i) no clear focus on the sample of choice (n = 2); (ii) secondary data (n = 1); and (iii) carried out in other countries in sub-Saharan Africa outside of South Africa (n = 4). Finally, 36 studies that assessed the prevalence of *Cryptosporidium* spp. in animal and human feces were included for quantitative synthesis (Figure 1).

### 3.2. Characteristics of Eligible Studies

With respect to the animal studies, 10 studies in total were included in the meta-analysis. These studies were published between the years 2008 and 2014 on the prevalence of *Cryptosporidium* spp. in various animals, including buffaloes, cats, cattle, dogs, elephants, goats, impala and sheep in South Africa (Table 1). Almost all studies were from the northern region of South Africa, which included four provinces, namely, Gauteng (n = 1), Limpopo (n = 4), Mpumalanga (n = 4) and North West (n = 2) (Table 1). Individually, the prevalence ranged from 0.00% to 80.0% across the various provinces (Table 1), with the highest prevalence recorded from the North West province and the lowest from the Mpumalanga province.

Additionally, 27 studies focusing on humans published between 1986 and 2020 were included in the meta-analysis on the prevalence of *Cryptosporidium* spp. in humans. These studies were from both the northern and southern regions of South Africa. Studies from the northern region included the Gauteng (n = 7), Limpopo (n = 6), Mpumalanga (n = 1), North West (n = 1) provinces, while the southern region included the Eastern Cape (n = 6) and KwaZulu-Natal (n = 8) provinces (Table 2). *Cryptosporidium* spp. prevalence for all the different provinces ranged from 2.89% to 72.94%, respectively (Table 2), with the highest pooled prevalence from the KwaZulu-Natal province and the lowest from the Gauteng province.

### 3.3. Pooling, Heterogeneity and Subgroup Analysis

#### 3.3.1. Prevalence in Animals Based on Hosts, Study Years and *Cryptosporidium* Species

Studies examining the prevalence of *Cryptosporidium* in animals found high heterogeneity based on the host, year of study and *Cryptosporidium* species (Table 3). In total, 2579 samples were screened, of which 374 tested positive to various species of *Cryptosporidium* spp. with a pooled prevalence estimate (PPE) of 21.5% (95%CI: 10.5–39.2%; *Q* = 391.34; *I*^2^ = 97.70; *Q–p* = 0.0003) (Table 3).

**Figure 2:**
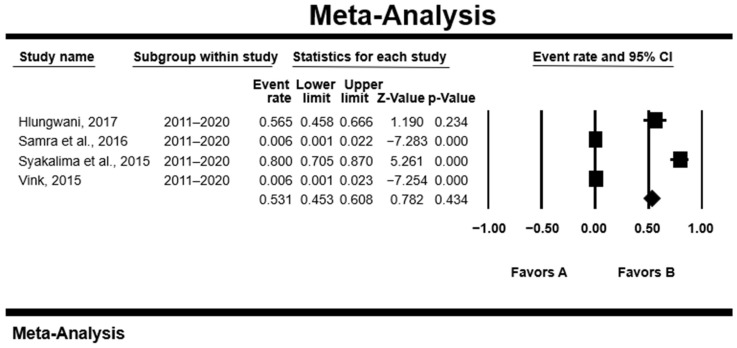
Forest plot of the prevalence of *Cryptosporidium* spp. from animal studies conducted during 2001–2010. The squares demonstrate the individual point estimates. The diamond at the base indicates the pooled estimate from the overall studies [30,36,37,38].

Animal studies conducted during the 2001–2010 duration had a slightly higher PPE of [11.7% (95%CI: 4.4–27.5); *Q* = 63.94; *I*^2^ = 95.31; *Q–p* = 0.0001] than those of the 2011–2020 duration [11.3% (95%CI: 1.1–58.8%); *Q* = 134.15, *I*^2^ = 97.76; *Q–p* = 0.0001] (Figure 2).

With references to species, *C. parvum* had the highest PPE of [3.7% (95%CI: 1.1–12.0%); Q = 17.58; *I*^2^ = 88.62, *Q–p* = 0.000], followed by *C. andersoni* [1.5% (95CI: 0.6–3.9%); *Q* = 0.86; *I*^2^ = 0.00; *Q–p* = 0.352] and *C. ubiquitum* 1.4%, and *C. bovis* had the lowest PPE [1.0% (95%CI: 0.4–2.3%); *Q* = 0.98; *I*^2^ = 0.00; *Q–p* = 0.320] (Table 3). The prevalence of *Cryptosporidium* spp. by animal host varied as the following: sheep had the highest PPE [31.5% (95%CI: 22.7–41.9%); *Q* = 0.64; *I*^2^ = 0.00; *Q–p* = 0.425], followed by goats [31.3% (95%CI: 11.2–62.0%); *Q* = 6.94; *I*^2^ = 85.60; *Q–p* = 0.008], dogs [30.4% (95%CI: 9.7–64.1%); *Q* = 30.4; *I*^2^ = 64.84; *Q–p* = 0.092], cattle [11.4% (95%CI 4.7–25.1%); *Q* = 49.31; *I*^2^ = 91.89; *Q–p* < 0.000], elephants [5.9% (95%CI: 0.1–73.8%); *Q* = 7.48; *I*^2^ = 86.62; *Q–p* = 0.006] and buffaloes [4.9% (95%CI; 2.7–8.9%); *Q* = 0.82; *I*^2^ = 0.00; *Q–p* = 0.36], and the lowest was for impala with a PPE of [3.9% (95%CI: 2.1–7.4%); *Q* = 0.31; *I*^2^ = 0.00; *Q–p* = 0.581] (Table 3).

#### 3.3.2. Assessment of Publication Bias in Animals

**Figure 3:**
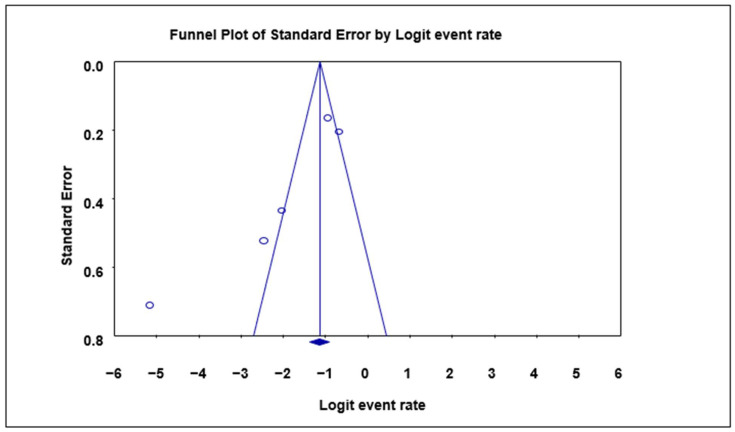
Funnel plot with 95% confidence limits of *Cryptosporidium* spp. pooled prevalence estimates of cattle subgroup studies that tested positive for *Cryptosporidium* species. The diamond at the base indicates the pooled estimate from the studies overall.

A funnel plot of standard error by logit event rate was used to ascertain the presence of publication bias in the eligible studies. No significant bias was observed overall in the animal studies using the Begg and Mazumdar rank correlation test except for the subgroup analysis, where with respect to the prevalence in cattle, significant bias was observed as evident by the asymmetry of the plot with a *p*-value of 0.0432 (Table 3; Figure 3).

#### 3.3.3. Prevalence in Humans Based on Study Years, Areas, Ages, HIV Statuses and Diagnostic Techniques

High heterogeneity was observed in studies looking at the prevalence of *Cryptosporidium* in humans depending on factors like age, HIV status, area, year of study and diagnostic method (Table 4). The 27 eligible studies for the evaluation of the prevalence of *Cryptosporidium* spp. in humans was conducted with data of studies published from 1983 to 2018. A total of 22,994 human fecal samples were examined, of which 3589 samples tested positive for *Cryptosporidium* spp., with a PPE of 18.1% (95%CI: 11.8–26.6). Substantial heterogeneity was observed [*Q* = 3655.54; *I*^2^ = 99.23; *Q–p* = 0.000] (Table 4).

The southern region had the highest PPE [19.8% (95%CI: 11.8–31.9%); *Q* = 1190.73, *I*^2^ = 98.91, *Q–p* = 0.000] compared to the northern region [16.9% (95%CI: 8.7–30.3%), *Q* = 1578.25, *I*^2^ = 99.11, *Q–p* = 0.000], with the highest PPE from the KwaZulu-Natal province (Table 4). Also, studies conducted during the 2001–2010 duration had the highest PPE, while studies conducted between 2011 and 2020 had the lowest [11.2% (95%CI: 5.7–21.0%); *Q* = 366.02; *I*^2^ = 98.36; *Q–p* = 0.000]. Despite the 1981–1990 period having had the highest number of studies and sample size, we observed a low PPE of 9.2% [95%CI: 4.9–16.4%; *Q* = 479.10; *I*^2^ = 98.54; *Q–p* = 0.000] (Table 4). With reference to species, *C. parvum* had the highest PPE of 18.3% [95%CI: 5.3–47.0%; *Q* = 223.28; *I*^2^ = 98.66; *Q–p* = 0.000] while *C. meleagridis* had the lowest PPE of 0.4% [95%CI: 0.1–1.6%; *Q* = 0.64; *I*^2^ = 0.00; *Q–p* = 0.424] (Table 4).

The age interval of 26–45 years had the highest PPE at 30.0% [*Q* = 91.33; 95%CI 14.1–52.9; *I*^2^ = 92.34; *Q–p* = 0.000], while the lowest was in the >45 yrs age interval at 24.2% [95%CI: 9.1–50.5%; *Q* = 40.44; *I*^2^ = 82.69; *Q–p* = 0.000] (Table 4).

**Figure 4:**
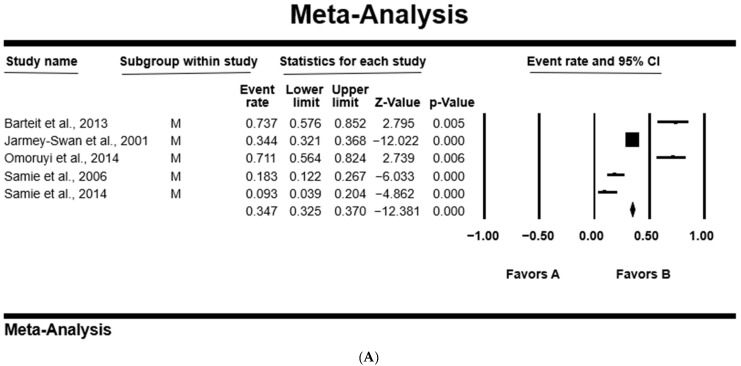

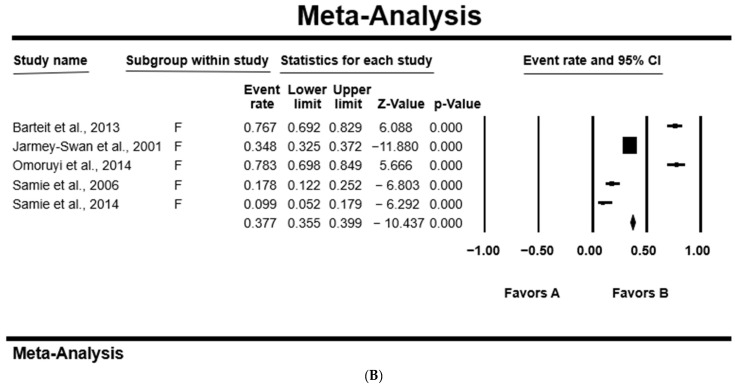
Forest plot showing the pooled estimates of *Cryptosporidium* spp. from studies conducted on (**A**) males and (**B**) females. The squares demonstrate the individual point estimates. The diamonds at the base indicate the pooled estimates from the overall studies [40,47,52,53,57].

In all studies, the PPE was higher in females at 41.1% [95%CI: 19.5–66.7%; *Q* = 187.25; *I*^2^ = 97.86; *Q–p* = 0.000], than 38.1% [95%CI: 20.5–59.6%; *Q* = 66.58; *I*^2^ = 93.99; *Q–p* = 0.000] in male participants (Table 4). Figure 4 shows a forest plot of individual point estimates for the combined prevalence estimates of males (A) and females (B).

With regards to HIV infection, the HIV-positive (HIV+) population had a comparatively higher PPE at 59.3% [95%CI: 19.8–89.6%; *Q* = 32.72; *I*^2^ = 93.89; *Q–p* = 0.000] as compared to 39.8% (95%CI: 12.3–75.8%); *Q* = 83.34; *I*^2^ = 97.60; *Q–p* = 0.000] in the HIV-negative (HIV–) population (Table 4).

*Cryptosporidium* spp. infections was high in diarrheal patients with a PPE of 24.4% [95%CI: 9.4–50.3); *Q* = 70.81; *I*^2^ = 97.18; *Q–p* = 0.000], as compared to non-diarrheal patients at 21.7% [95%CI: 8.7–44.8%); *Q* = 45.30; *I*^2^ = 95.59; *Q–p* = 0.000] (Table 4). With respect to diagnostic techniques, our analyses showed that polymerase chain reaction–restriction fragment length polymorphism (PCR-RFLP) had the highest *Cryptosporidium* spp. detection sensitivity with a PPE at [77.8% (95%CI: 65.9–86.4%); *Q* = 0.60; *I*^2^ = 0.00; *Q–p* = 0.438], followed by ELISA [66.7% (95%CI 46.4–82.3%); *Q* = 101.98; *I*^2^ = 97.06; *Q–p* = 0.000], loop-mediated isothermal amplification (LAMP) [45.4% (95%CI: 26.6–56.6%); *Q* = 3.91; *I*^2^ = 74.39; *Q–p* = 0.048], PCR [25.3% (95%CI: 11.5–46.9%); *Q* = 203.65; *I*^2^ = 96.07; *Q–p* = 0.000], quantitative polymerase chain reaction (qPCR) [20.7% (95%CI: 11.1–35.4%); *Q* = 30.50; *I*^2^ = 93.44; *Q–p* = 0.000], microscopy [10.1% (95%CI: 6.1–16.2%); *Q* = 1761.79; *I*^2^ = 98.87; *Q–p* = 0.000] and immunofluorescence antibody test (IFAT) [8.4% (95%CI: 0.7–53.2%); *Q* = 44.89; *I*^2^ = 97.77; *Q–p* = 0.000], and rapid diagnostic test (RDT) had the lowest PPE [7.9% (95%CI: 3.2–18.0%); *Q* = 143.71; *I*^2^ = 97.91; *Q–p* = 0.000] (Table 4).

### 3.4. Publication Bias Assessment in Human Studies

**Figure 5:**
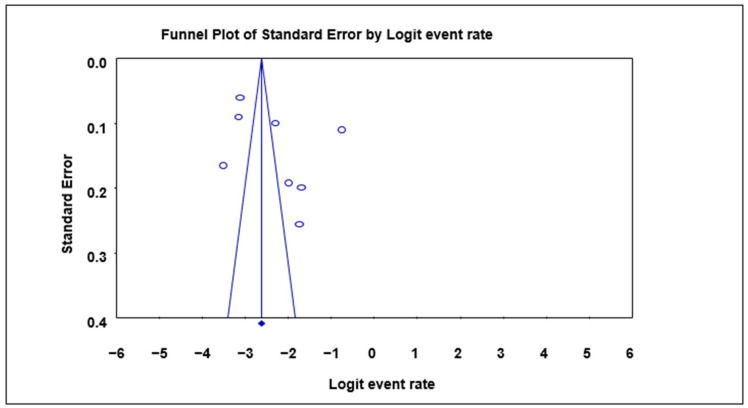
Funnel plot with 95% confidence limits of *Cryptosporidium* spp. pooled prevalence estimates of 1981–1990 interval subgroup studies that tested positive for *Cryptosporidium* spp. in humans. The diamond at the base indicates the pooled estimate from the studies overall.

The Begg and Mazumdar rank correlation test revealed no significant publication bias for almost all the parameters except for the study year period 1981–1990, where significant bias was observed of both the asymmetry of the funnel plots and *p*-value of 0.042 (Table 4; Figure 5).

## 4. Discussion

This study recorded an overall PPE of 21.5% for *Cryptosporidium* spp. infection in animals. Similar findings have also been reported in Australia (22.3%) and Tunisia (18.9%) [5,64]. On the other hand, higher prevalence above 50.0% was reported in an animal study from China and in a global review of *Cryptosporidium* spp. [65,66]. Most of the animal studies included in this meta-analysis were focused on the northern region of South Africa, where our analysis recorded a PPE of 13.7% of *Cryptosporidium* spp. infections. Data of animal studies were scarce in the southern regions; this paucity of studies may be due to a low research interest, as cryptosporidiosis is possibly not regarded as problematic for livestock. Furthermore, the results indicated a declining trend of *Cryptosporidium* spp. infection prevalence overtime, whereby the 2011–2020 period had a slightly lower pooled estimated prevalence as compared to the 2001–2010 period of study. This observation could be associated with the use of proper sanitary toilets, medication and improved animal husbandry practice. These observations are in accordance with the World Health Organization (WHO) and Global Roadmap 2012 mandate to decrease the prevalence of zoonotic diseases by 2020 through the improvement in veterinary public health practice and a focus on the supply of safe and palatable water, good sanitary infrastructures and proper hygiene practice [67]. Moreover, treatment, sanitation and proper hygiene practices have been proven to assist in reducing the prevalence of *Cryptosporidium* spp. infections in livestock [68,69,70].

Among all *Cryptosporidium* spp. observed in this study, *C. parvum* (3.7%) had the highest PPE, followed by *C. bovis* (1.0%). Similar findings have been reported in Greece and Peru, whereby *C. parvum* (64.3%) was more prevalent in comparison to both *C. andersoni* + *C. bovis* (7.1%) prevalence in animals [71,72]. In contrast, Ref. [73] reported different findings, whereby *C. bovis* (57.0%) was the most common *Cryptosporidium* spp. of health concern and *C. parvum* (7.0%), the species of least concern in veterinary medicine.

Animal hosts facilitate the spread of *Cryptosporidium* spp. differently according to their level of relationship with humans. This meta-analysis of pooled data indicated that there were more cattle studies as compared to other animal hosts. Sheep had the highest PPE (31.5%) as compared to cattle (11.4%). Similar results have been reported in India, where sheep had a 35.0% prevalence compared to cattle, of 5.0% prevalence [74]. Additionally, Odenrian and Ademola [75] observed that cattle (26.1%) in Nigeria appeared to be more exposed to *Cryptosporidium* spp. infections as compared to other domestic animals.

We observed an overall PPE of *Cryptosporidium* spp. of 18.1% in our present human study. Similarly, this result has been reported in studies from the southern region (20.0%), the Oromia (18.0%) province in Ethiopia and in humans from southern Africa (6.8%) [76,77]. Urban areas are known to have access to potable water with better sanitation practices, which can lower the spread of cryptosporidiosis [78]. In the current study, the northern region of South Africa had a lower prevalence (16.9%) compared to the southern region (19.8%), which we believe is due to better sanitary practices in the north than the resource-poor southern region. This is in accordance with the findings reported by Kalantari et al. [79]. Our analysis indicated an increase in the prevalence of *Cryptosporidium* spp. with decreasing sample size. This accounted for the increase in prevalence from 1981 to 2010 and then a decline in the 2011–2020 period, which could have been due to an increase in the population sample size, which agrees with the findings in Ethiopia [80].

All characterized *Cryptosporidium* spp. were detected by serological and molecular techniques and our findings showed *C. parvum* to be the most abundant species infecting humans. These findings are in agreement with the findings obtained in Iran, whereby *C. parvum* (84.4%) was the most prevalent species followed by *C. hominis* (13.4%) [79]. However, other studies have reported *C. hominis* as the most common *Cryptosporidium* spp. that infected humans followed by *C. parvum* in Malawi and India [81,82]. The present study observed a higher PPE for the population group aged 26–45 years, followed by <6 months–25 years and the lowest in the >45 years age group. Similar results were obtained whereby the 1–25 years (3.0%; 15.4%) group had high *Cryptosporidium* spp. prevalence as compared to the >45 years group (0.4%; 4.0%) in studies from Iran and Scotland [83,84]. Interestingly, the literature generally indicated that younger children were more susceptible to contracting *Cryptosporidium* spp. infections as compared to other older age groups [85]. This is because children tend to ignore hygiene while playing outside in grounds that might harbor zoonotic microorganisms of fecal origin [86]. Moreover, higher prevalence among children may reflect a lack of immunity as compared to older groups who acquire it due to exposure to *Cryptosporidium* spp. infection during their lifetime due to activities such as farming and swimming [87,88].

Our results revealed that females (41.1%) had a higher PPE compared to males (38.1%), which is consistent with previous reports from villages around Lake Atitlan, Guatemala, and in Delta State, Nigeria, where prevalences of 42.9% and 3.5% were found in females, respectively, and of 24.1%, and 2.1% in males, respectively [88,89]. However, higher prevalence was observed in males (13.0% and 13.3%) compared to females (6.1% and 7.1%) as recorded in Zambia and Pakistan, respectively [90,91]. This high prevalence in females could be associated with a lack of access to clean water, participation in day care, conducting house chores including cleaning and washing clothes and sometimes bad sanitary activities due to socioeconomic conditions [92]. Historically, it is rare for males to consult health practitioners whenever they are ill, and they will either try home remedies, while females consult practitioners for most health complications happening in their bodies, and hence, they appear to have higher records of infection [93,94].

Undoubtably, available data have shown that the *Cryptosporidium* parasite is an opportunistic infection, particularly in immunosuppressed individuals [95]. Our results indicated that HIV+ individuals (59.3%) were more exposed to infection with *Cryptosporidium* spp. as compared to HIV− individuals (39.8%). The peak occurrence of *Cryptosporidium* spp. in HIV+ individuals was consistent with previous observations from Uganda, which had 73.6% and 5.9% HIV+ and HIV− individuals, respectively [96]. Our findings appeared to be higher as compared to those reported in Nigeria [97] and also fell within the epidemiological range of the world’s rate (0–78.1%) for *Cryptosporidium* spp. infections [98]. This relatively high PPE of *Cryptosporidium* spp. infection could be linked to poor hygiene practices, water scarcity, close contact with animals and high rate of immunocompromised individuals [24,99].

Numerous diagnostic methods can be used to detect *Cryptosporidium* infection in humans and animals worldwide including histology, immunology, microscopy and molecular techniques [100]. The findings from this study suggest that the frequently applied diagnostic method for *Cryptosporidium* species in South Africa was microscopy, followed by PCR, RDT, ELISA, qPCR, IFAT and LAMP, and the least used was PCR-RFLP. This agrees with documented reports by Kalantari et al. [80] in Iran, where they reported microscopy as the most employed diagnostic approach for the detection of *Cryptosporidium* spp. infection. Mohebali et al. [81] detected similar *Cryptosporidium* prevalence (10%) in Ethiopia using a modified Ziehl–Neelsen staining diagnostic technique. This increase in the detection of *Cryptosporidium* spp. prevalence might be linked to the use of serological and molecular techniques, which are more sensitive and less time-consuming.

This study confirmed the prevalence of *Cryptosporidium* spp. in animals, humans and the environment (soil and water). Our results highlight the importance of “One Health” because *Cryptosporidium* spp. has been proven to exist in humans, animals and the environment. Future researchers should be encouraged to use the “One Health” approach to developing methods that explicitly examine the relationships between human–animal–environment frameworks, with a particular focus on *Cryptosporidium* infections.

## 5. Highlights and Limitations

This systematic review and meta-analysis study used good-quality studies to present a summary of unbiased results of both animal and human *Cryptosporidium* prevalence in South Africa and revealed that there are some provinces where *Cryptosporidium* spp. infections have not yet been studied. With respect to humans, there are no studies published in the Northern Cape, Western Cape and Free State provinces, while for animals, there are no published studies in the KwaZulu-Natal, Northern Cape, Western Cape and Eastern Cape provinces. Additionally, this study demonstrated the impact of the domestic–wildlife–human interface on the prevalence of *Cryptosporidium* spp. infection and distribution, which emphasizes the need for studies focusing on a “One Health” approach to produce multi-data covering animal and human hosts as well as the environment, such as contaminated water and soil.

It must be noted that this systematic review and meta-analysis had some limitations. The study by Lukasova et al. [34] had a very small number of samples in the Gauteng (n = 1) and North West (n = 8) provinces, which were examined for the presence of *Cryptosporidium* spp., and there was also lack of similar studies in some provinces (e.g., the Northern Cape province with zero publications/representation in both animal and human studies). Additionally, the majority of studies included in this meta-analysis were conducted using microscopic diagnostic techniques, which have a lesser diagnostic sensitivity as compared to molecular and immunological techniques. Moreover, there was no repeated fecal sample examination conducted, which might have resulted in possible false-positive or -negative results. This means that the reported prevalence might have been underestimated.

Due to the small number of studies on some subgroups such as (i) various *Cryptosporidium* species such as *C. andersoni, C. bovis, C. ubiquitum*, *C. hominis* and *C. muris;* (ii) various hosts such as buffaloes, dogs, elephants, goats, sheep and impala; (iii) the wide use of various diagnostic methods such as IFAT, LAMP and PCR-RFLP; and (iv) the lack of studies in the period 1991–2000, the identified formal assessment of publication bias using funnel plots and Begg’s rank and Mazumdar test was not possible. However, meta-analyses that include fewer than 10 studies or have a high degree of heterogeneity between studies may lead to misleading results from these assessment tools. When there is a high level of heterogeneity, it is very difficult to evaluate the actual results of statistically significant publication bias tests. Because there is high heterogeneity across analyses, readers should exercise caution when interpreting pooled analyses and subgroups.

## 6. Conclusions

The data generated in this study indicated that the prevalence of *Cryptosporidium* spp. infections was slightly higher in animal than human hosts in South Africa. However, we further observed that there was a lack of *Cryptosporidium* spp. prevalence studies for both animals and humans in some of the provinces. Furthermore, human infections were prevalent in HIV+ and immunocompromised patients, emphasizing that they were a high-risk group for opportunistic diseases such as cryptosporidiosis. However, the results of the included studies varied greatly, between their sampling methods, sample sizes, study locations and diagnostic techniques used, and this needs to be taken into account and may explain some of the inconsistencies. The occurrence and prevalence of *Cryptosporidium* spp. infections in animals is of public health importance, hence, more studies involving both domestic and wild animals are required. The findings of this study suggest the necessity for a “One Health” strategy to promote public hygiene, animal husbandry and regular screening for *Cryptosporidium* spp. infections of animals, humans and the environment (soil and water) in all nine provinces of South Africa.

## Figures and Tables

**Table 1:** List and characteristics of eligible studies included in the meta-analysis with respect to animal study prevalence by different provinces in South Africa.

Study Authors	Animal Host (n)	Study Area (Province)	Sample Size	No. of Positives	Prevalence (%)
Bakheit et al. [29]	Cattle (n = 107) Horse (n = 78) Sheep (n = 85)	Free State	270	79	29.26
Hlungwani [30]	Cattle (n = 52) Goat (n = 33)	Limpopo	85	48	56.47
Lukášová et al. [31]	Cat (n = 1)	Gauteng	1	0	0.00
Lukášová et al. [31]	African civet (n = 2) African wild cat (n = 1) Banded mongoose (n = 3) Black-backed jackal (n = 1) Caracal (n = 2) Cat (n = 1) Cheetah (n = 4) Dog (n = 11) Lion (n = 13) Serval (n = 2) Spotted hyena (n = 3) Striped polecat (n = 2)	Limpopo	45	7	15.56
Lukášová et al. [31]	Bat-eared fox (n = 1) Black-backed jackal (n = 6) Caracal (n = 1)	North West	8	0	0.00
Samie et al. [32]	Cat (n = 25) Dog (n = 25)	Limpopo	50	19	38.00
Samie et al. [33]	Chicken (n = 28) Goat (n = 93) Sheep (n = 4) Cattle (n = 187)	Limpopo	312	98	31.41
Samra et al. [34]	Buffalo (n = 141) Elephant (n = 144) Impala (n = 161)	Mpumalanga	446	29	6.50
Samra et al. [35]	Buffalo (n = 71) Elephant (n = 72) Impala (n = 71)	Mpumalanga	214	79	36.92
Samra et al. [36]	Calf (Bovine) (n = 352)	Mpumalanga	352	2	7.95
Syakalima et al. [37]	Pig (n = 90)	North West	90	72	80.00
Vink [38]	Calf (Bovine) (n = 345)	Mpumalanga	345	2	0.58

**Table 2:** List and characteristics of eligible studies included in the meta-analysis with respect to human study prevalence by different provinces in South Africa.

Study Authors	Study Area (Province)	Sample Size	No. of Positives	Prevalence (%)
Abebe et al. [39]	Limpopo	84	25	29.76
Bartelt et al. [40]	Limpopo	251	165	65.74
Becker et al. [41]	Eastern Cape	1428	164	11.49
Berkowitz et al. [42]	Gauteng	121	18	14.88
Etinosa [43]	Eastern Cape	180	122	67.78
Fripp et al. [44]	Gauteng	6870	289	4.21
Geyer et al. [45]	Gauteng	78	20	25.64
Hlungwani [30]	Gauteng	362	80	22.10
Hlungwani [30]	Limpopo	218	159	72.94
Htun et al. [46]	Eastern Cape	842	179	21.26
Jarmey-Swan et al. [47]	KwaZulu-Natal	2800	1300	46.43
Leav et al. [48]	KwaZulu-Natal	101	25	24.75
Moodley et al. [49]	KwaZulu-Natal	1229	111	9.03
Msolo et al. [50]	Eastern Cape	53	3	5.66
Müller et al. [51]	Eastern Cape	934	28	2.99
Omoruyi et al. [52]	Eastern Cape	180	47	26.11
Samie et al. [53]	Limpopo	244	44	18.03
Samie et al. [54]	Limpopo	255	46	18.04
Samie et al. [55]	Limpopo	528	143	27.08
Samie et al. [56]	Limpopo	322	42	13.04
Samie et al. [57]	Limpopo	151	15	9.93
Samra et al. [58]	Gauteng	141	25	17.73
Samra et al. [58]	Mpumalanga	128	11	8.59
Samra et al. [58]	North West	147	14	9.52
Samra et al. [58]	KwaZulu-Natal	26	4	15.39
Smith and Van den Ende [59]	KwaZulu-Natal	259	31	11.97
Steele et al. [60]	Gauteng	1316	38	2.89
Steele et al. [14]	Gauteng	3186	129	4.05
Trönnberg et al. [61]	KwaZulu-Natal	120	25	20.83
Walters [62]	KwaZulu-Natal	91	53	58.24
Witienberg et al. [63]	KwaZulu-Natal	194	30	15.46

**Table 3:** Pooled prevalence estimates and risk factors associated with *Cryptosporidium* species infection in animals.

Risk Factor	No. of Studies	Pooled Prevalence Estimates			Measure of Heterogeneity		*Q–P*	Publication Bias
		Sample Size	No. of Positives	Prevalence 95%CI (%)	*Q*	*I^2^*		Begg and Mazumdar Rank *p*-Value
Overall animals	10	2579	374	21.5 (10.5–39.2)	391.34	97.70	0.000	0.123
Study year								
2001–2010	4	1015	102	11.7 (4.4–27.5)	63.94	95.31	0.000	0.500
2011–2020	4	872	124	11.3 (1.1–58.8)	134.15	97.76	0.000	0.248
*Cryptosporidium* species								
*C. andersoni*	2	299	4	1.5 (0.6–3.9)	0.86	0.00	0.352	–
*C. bovis*	2	566	5	1.0 (0.4–2.3)	0.98	0.00	0.320	
*C. parvum*	3	882	32	3.7 (1.1–12.0)	17.58	88.62	0.000	0.301
*C. ubiquitum*	1	214	3	1.4				–
Animal species								
Buffalo	2	212	10	4.9 (2.7–8.9)	0.82	0.00	0.364	–
Cattle	5	749	100	11.4 (4.7–25.1)	49.31	91.89	0.000	0.025
Dog	2	38	13	30.4 (9.7–64.1)	2.84	64.84	0.092	–
Elephant	2	216	37	5.9 (0.1–73.8)	7.48	86.62	0.006	–
Goat	2	126	48	31.3 (11.2–62.0)	6.94	85.60	0.008	–
Impala	2	232	9	3.9 (2.1–7.4)	0.31	0.00	0.581	–
Sheep	2	89	28	31.5 (22.7–41.9)	0.64	0.00	0.425	–

**Table 4:** Pooled prevalence estimates and risk factors associated with *Cryptosporidium* species infection in humans.

Risk Factor	No. of Studies	Pooled Prevalence Estimates			Measure of Heterogeneity		*Q–P*	Publication Bias
		Sample Size	No. of Positives	Prevalence 95%CI (%)	*Q*	*I* ^2^		Begg and Mazumdar Rank (*p*-Value)
Overall humans	27	22,787	3510	18.1 (11.8–26.6)	3655.54	99.23	0.000	0.411
Study region								
Northern region	15	13,825	1175	16.9 (8.7–30.3)	1578.25	99.11	0.000	0.200
Southern region	14	8437	2122	19.8 (11.8–31.4)	1190.73	98.91	0.000	0.274
Sex								
Female	5	2067	785	41.1 (19.5–66.7)	187.25	97.86	0.000	0.500
Male	5	1786	615	38.1 (20.5–59.6)	66.58	93.99	0.000	0.500
Age								
<6 months–25 years	19	3636	1214	28.7 (23.3–34.7)	112.60	84.01	0.000	0.376
26–45 years	8	369	130	30.0 (14.1–52.9)	91.33	92.34	0.000	0.310
>45 years	8	153	43	24.2 (9.1–50.5)	40.44	82.69	0.000	0.310
Diagnostic technique								
Microscopy	21	25,475	1570	10.1 (6.1–16.2)	1761.79	98.87	0.000	0.359
ELISA	4	1081	598	66.7 (46.4–82.3)	101.98	97.06	0.000	0.087
IFAT	2	546	36	8.4 (0.7–53.2)	44.89	97.77	0.000	–
LAMP	2	237	93	45.4 (26.6–56.6)	3.91	74.39	0.048	–
RDT	4	3257	331	7.9 (3.2–18.0)	143.71	97.91	0.000	0.249
qPCR	3	717	139	20.7 (11.1–35.4)	30.50	93.44	0.000	0.059
PCR	9	991	252	25.3 (11.5–46.9)	203.65	96.07	0.000	0.500
PCR-RFLP	2	64	50	77.8 (65.9–86.4)	0.60	0.00	0.438	–
Study year								
1981–1990	8	13,557	768	9.2 (4.9–16.4)	479.10	98.54	0.000	0.042
1991–2000	2	2901	1325	35.4 (17.5–58.6)	17.19	94.18	0.000	–
2001–2010	5	1223	502	42.7 (24.4–63.2)	167.58	97.61	0.000	0.500
2011–2020	7	4422	675	11.2 (5.7–21.0)	366.02	98.36	0.000	0.440
*Cryptosporidium* species								
*C. hominis*	3	1165	46	4.0 (3.0–5.3)	1.43	0.000	0.489	0.301
*C. meleagridis*	2	585	2	0.4 (0.1–1.6)	0.64	0.00	0.424	–
*C. muris*	1	580	1	–	–	–	–	–
*C. parvum*	4	1636	403	18.3 (5.3–47.0)	223.28	98.66	0.000	0.500
HIV status								
HIV+	3	259	180	59.3 (19.8–89.6)	32.72	93.89	0.000	0.301
HIV−	3	396	149	39.8 (12.3–75.8)	83.34	97.60	0.000	0.059
Fecal consistency								
Diarrhea	3	691	243	24.4 (9.4–50.3)	70.81	97.18	0.000	0.301
Non-diarrhea	3	565	148	21.7 (8.7–44.8)	45.30	95.59	0.000	0.301

ELISA, enzyme-linked immunosorbent assay; HIV, human immunodeficiency virus; IFAT, immunofluorescence antibody test; qPCR, quantitative polymerase chain reaction; PCR, polymerase chain reaction; LAMP, loop-mediated isothermal amplification; PCR-RFLP, polymerase chain reaction–restriction fragment length polymorphism.

## Data Availability

The datasets generated and/or analyzed during the current study are available from the corresponding author on reasonable request.

## References

[1] Burns J.K. (2011). The mental health gap in South Africa: A human rights issue. Equal. Rights Rev..

[2] Stiegler N., Bouchard J.P. (2020). South Africa: Challenges and successes of the COVID-19 lockdown. Ann. Médico-Psychol..

[3] Hammond-Aryee K., Esser M., Van Helden P.D. (2014). Toxoplasma gondii sero-prevalence studies on humans and animals in Africa. S. Afr. Fam. Pract..

[4] Abubakar I., Aliyu S.H., Arumugam C., Usman N.K., Hunter P.R. (2007). Treatment of cryptosporidiosis in immunocompromised individuals: Systematic review and meta-analysis. Br. J. Clin. Pharmacol..

[5] Hatam-Nahavandi K., Ahmadpour E., Carmena D., Spotin A., Bangoura B., Xiao L. (2019). *Cryptosporidium* infections in terrestrial ungulates with focus on livestock: A systematic review and meta-analysis. Parasit. Vectors.

[6] Brook E., Hart C.A., French N., Christley R. (2008). Prevalence and risk factors for *Cryptosporidium* spp. infection in young calves. Vet. Parasitol..

[7] Gambhir I.S., Jaiswal J.P., Nath G. (2003). Significance of *Cryptosporidium* as an aetiology of acute infectious diarrhoea in elderly Indians. Trop. Med. Int. Health.

[8] Hawker J., White J., Catchpole M. (2000). Quarterly communicable disease review April to June 2000. J. Public Health.

[9] Khalil I.A., Troeger C., Rao P.C., Blacker B.F., Brown A., Brewer T.G., Colombara D.V., De Hostos E.L., Engmann C., Guerrant R.L. (2018). Morbidity, mortality, and long-term consequences associated with diarrhoea from *Cryptosporidium* infection in children younger than 5 years: A meta-analyses study. Lancet Glob. Health.

[10] Bouzid M., Steverding D., Tyler K.M. (2008). Detection and surveillance of waterborne protozoan parasites. Curr. Opin. Biotechnol..

[11] Chalmers R.M., Campbell B., Crouch N., Davies A.P. (2010). Clinical laboratory practices for detection and reporting of *Cryptosporidium* in community cases of diarrhoea in the United Kingdom, 2008. Eurosurveillance.

[12] Naciri M., Lefay M.P., Mancassola R., Poirier P., Chermette R. (1999). Role of *Cryptosporidium* parvum as a pathogen in neonatal diarrhoea complex in suckling and dairy calves in France. Vet. Parasitol..

[13] Sow S.O., Muhsen K., Nasrin D., Blackwelder W.C., Wu Y., Farag T.H., Saha D. (2016). The burden of *Cryptosporidium* diarrheal disease among children< 24 months of age in moderate/high mortality regions of sub-Saharan Africa and South Asia, utilizing data from the Global Enteric Multicenter Study (GEMS). PLoS Negl. Trop. Dis..

[14] Steele A.D., Gove E., Meewes P.J. (1989). Cryptosporidiosis in white patients in South Africa. J. Infect..

[15] Hunter P.R., Thompson R.A. (2005). The zoonotic transmission of Giardia and *Cryptosporidium*. Int. J. Parasitol..

[16] Xiao L., Fayer R., Ryan U., Upton S.J. (2004). *Cryptosporidium* taxonomy: Recent advances and implications for public health. Clin. Microbiol. Rev..

[17] Wang R., Wang H., Sun Y., Zhang L., Jian F., Qi M., Xiao L. (2011). Characteristics of *Cryptosporidium* transmission in pre-weaned dairy cattle in Henan, China. J. Clin. Microbiol..

[18] Vermeulen L.C., Benders J., Medema G., Hofstra N. (2017). Global *Cryptosporidium* loads from livestock manure. Environ. Sci. Technol..

[19] Oates S.C., Miller M.A., Hardin D., Conrad P.A., Melli A., Jessup D.A., Miller W.A. (2012). Prevalence, environmental loading, and molecular characterization of *Cryptosporidium* and Giardia isolates from domestic and wild animals along the Central California Coast. Appl. Environ. Microbiol..

[20] Silverlås C., Bosaeus-Reineck H., Näslund K., Björkman C. (2013). Is there a need for improved *Cryptosporidium* diagnostics in Swedish calves?. Int. J. Parasitol..

[21] Efstratiou A., Ongerth J., Karanis P. (2017). Evolution of monitoring for Giardia and *Cryptosporidium* in water. Water Res..

[22] Ryan U., Hijjawi N., Xiao L. (2018). Foodborne cryptosporidiosis. Inter. J. Parasitol..

[23] Havelaar A.H., Melse J.M. (2003). Quantifying Public Health Risk in the WHO Guidelines for Drinking-Water Quality: A Burden of Disease Approach.

[24] Shaposhnik E.G., Abozaid S., Grossman T., Marva E., On A., Azrad M., Peretz A. (2019). The prevalence of *Cryptosporidium* among children hospitalized because of gastrointestinal symptoms and the efficiency of diagnostic methods for *Cryptosporidium*. Am. J. Trop. Med. Hyg..

[25] Omolabi K.F., Odeniran P.O., Soliman M.E. (2022). A meta-analysis of *Cryptosporidium* species in humans from southern Africa (2000–2020). J. Parasit. Dis..

[26] Ramatla T., Tawana M., Lekota K.E., Thekisoe O. (2023). Antimicrobial resistance genes of Escherichia coli, a bacterium of “One Health” importance in South Africa: Systematic review and meta-analysis. AIMS Microbiol..

[27] Tawana M., Onyiche T.E., Ramatla T., Thekisoe O. (2023). A “One Health” perspective of Africa-wide distribution and prevalence of Giardia species in humans, animals and waterbodies: A systematic review and meta-analysis. Parasitology.

[28] Ramatla T., Tawana M., Mphuthi M.B., Onyiche T.E., Lekota K.E., Monyam M.C., Ndou R., Bezuidenhout C., Thekisoe O. (2022). Prevalence and antimicrobial resistance profiles of Campylobacter species in South Africa: A “One Health” approach using systematic review and meta-analysis. Int. J. Infect. Dis..

[29] Bakheit M.A., Torra D., Palomino L.A., Thekisoe O.M., Mbati P.A., Ongerth J., Karanis P. (2008). Sensitive and specific detection of *Cryptosporidium* species in PCR-negative samples by loop-mediated isothermal DNA amplification and confirmation of generated LAMP products by sequencing. Vet. Parasitol..

[30] Hlungwani H.A. (2017). Molecular Detection and Identification of *Cryptosporidium* Species Isolated from Human and Animal Sources in Limpopo and Gauteng Provinces. Doctoral Dissertation.

[31] Lukášová R., Halajian A., Bártová E., Kobédová K., Swanepoel L.H., O’Riain M.J. (2018). The occurrence of some nonblood protozoan parasites in wild and domestic mammals in South Africa. J. Wildl. Dis..

[32] Samie A., Tsipa M.A., Bessong P. (2013). The epidemiology of *Cryptosporidium* in cats and dogs in the Thohoyandou region, South Africa. Afr. J. Microbiol. Res..

[33] Samie A., Hlungwani A.H., Mbati P.A. (2017). Prevalence and risk factors of *Cryptosporidium* species among domestic animals in rural communities in Northern South Africa. Trop. Biomed..

[34] Samra N.A., Jori F., Samie A., Thompson P. (2011). The prevalence of *Cryptosporidium* spp. oocysts in wild mammals in the Kruger National Park, South Africa. Vet. Parasitol..

[35] Samra N.A., Thompson P.N., Jori F., Frean J., Poonsamy B., Du Plessis D., Mogoye B., Xiao L. (2013). Genetic characterization of *Cryptosporidium* spp. in diarrhoeic children from four provinces in South Africa. Zoonoses Public Health.

[36] Samra N.A., Jori F., Cacciò S.M., Frean J., Poonsamy B., Thompson P.N. (2016). *Cryptosporidium* genotypes in children and calves living at the wildlife or livestock interface of the Kruger National Park, South Africa. Onderstepoort J. Vet. Res..

[37] Syakalima M., Noinyane M.I., Ramaili T., Motsei L., Nyirenda M. (2015). A coprological assessment of cryptosporidiosis and giardiosis in pigs of Mafikeng villages, North West province of South Africa. Indian J. Anim. Res..

[38] Vink J.J.W.G. (2015). Cryptosporidiosis in Pre-Weaned Calves at the Wildlife/Livestock Interface of the Kruger National Park, South Africa. Master’s Thesis.

[39] Abebe L.S., Smith J.A., Narkiewicz S., Oyanedel-Craver V., Conaway M., Singo A., Amidou S., Mojapelo P., Brant J., Dillingham R. (2014). Ceramic water filters impregnated with silver nanoparticles as a point-of-use water-treatment intervention for HIV-positive individuals in Limpopo Province, South Africa: A pilot study of technological performance and human health benefits. J. Water Health.

[40] Bartelt L.A., Sevilleja J.E., Barrett L.J., Warren C.A., Guerrant R.L., Bessong P.O., Dillingham R., Samie A. (2013). High anti-*Cryptosporidium* parvum IgG seroprevalence in HIV-infected adults in Limpopo, South Africa. Am. J. Trop. Med. Hyg..

[41] Becker S.L., Müller I., Mertens P., Herrmann M., Zondie L., Beyleveld L., Gerber M., du Randt R., Pühse U., Walter C. (2017). PCR-based verification of positive rapid diagnostic tests for intestinal protozoa infections with variable test band intensity. Acta Trop..

[42] Berkowitz F.E., Vallabh W., Buqwana A., Heney C. (1988). Cryptosporidiosis in black South African children. S. Afr. Med. J..

[43] Etinosa O.B. (2010). Immunological and Molecular Characterization of *Cryptosporidium* Species in HIV-Positive and HIV-Negative Diarrhoea Patients in the Nkonkobe Municipality of the Eastern Cape Province of South Africa: A Pilot Study. Doctoral Dissertation.

[44] Fripp P.J., Bothma M.T., Crewe-Brown H.H. (1991). Four years of cryptosporidiosis at GaRankuwa Hospital. J. Infect..

[45] Geyer A., Crewe-Brown H.H., Greeff A.S., Fripp P.J., Steele A.D., Van T.S., Clay C.G. (1993). The microbial aetiology of summer paediatric gastroenteritis at Ga-Rankuwa Hospital in South Africa. East. Afr. Med. J..

[46] Htun N.S.N., Odermatt P., Müller I., Yap P., Steinmann P., Schindler C., Gerber M., Du Randt R., Walter C., Pühse U. (2018). Association between gastrointestinal tract infections and glycated haemoglobin in school children of poor neighbourhoods in Port Elizabeth, South Africa. PLoS Negl. Trop. Dis..

[47] Jarmey-Swan C., Bailey I.W., Howgrave-Graham A.R. (2001). Ubiquity of the water-borne pathogens, *Cryptosporidium* and Giardia, in KwaZulu-Natal populations. Water S. Afr..

[48] Leav B.A., Mackay M.R., Anyanwu A., O’Connor R.M., Cevallos A.M., Kindra G., Rollins N.C., Bennish M.L., Nelson R.G., Ward H.D. (2002). Analysis of sequence diversity at the highly polymorphic Cpgp40/15 locus among *Cryptosporidium* isolates from human immunodeficiency virus-infected children in South Africa. Infect. Immun..

[49] Moodley D., Jackson T.F.H.G., Gathiram V., Van Den Ende J. (1991). *Cryptosporidium* infections in children in Durban Seasonal variation, age distribution and disease status. S. Afr. Med. J..

[50] Msolo L., Iweriebor B.C., Okoh A.I. (2020). Rotavirus and *Cryptosporidium* pathogens as etiological proxies of gastroenteritis in some pastoral communities of the Amathole District Municipality, Eastern Cape, South Africa. BMC Res. Notes.

[51] Müller I., Yap P., Steinmann P., Damons B.P., Schindler C., Seelig H., Htun N.S., Probst-Hensch N., Gerber M., du Randt R. (2016). Intestinal parasites, growth and physical fitness of schoolchildren in poor neighbourhoods of Port Elizabeth, South Africa: A cross-sectional survey. Parasit. Vectors.

[52] Omoruyi B.E., Nwodo U.U., Udem C.S., Okonkwo F.O. (2014). Comparative diagnostic techniques for *Cryptosporidium* infection. Molecules.

[53] Samie A., Bessong P.O., Obi C.L., Sevilleja J.E.A.D., Stroup S., Houpt E., Guerrant R.L. (2006). *Cryptosporidium* species: Preliminary descriptions of the prevalence and genotype distribution among school children and hospital patients in the Venda region, Limpopo Province, South Africa. Exp. Parasitol..

[54] Samie A., Obi C.L., Tzipori S., Weiss L.M., Guerrant R. (2007). Microsporidiosis in South Africa: PCR detection in stool samples of HIV-positive and HIV-negative individuals and school children in Vhembe district, Limpopo Province. Trans. R. Soc. Trop. Med. Hyg..

[55] Samie A., Guerrant R.L., Barrett L., Bessong P.O., Igumbor E.O., Obi C.L. (2009). Prevalence of intestinal parasitic and bacterial pathogens in diarrhoeal and non-diarroeal human stools from Vhembe district, South Africa. J. Health Popul. Nutr..

[56] Samie A., Bessong P.O., Obi C.L., Dillingham R., Guerrant R.L., Ar Zajae V. (2011). Bacterial and Parasitic Agents of Infectious Diarrhoea in the Era of HIV and AIDS-The Case of a Semi Rural Community in South Africa. Microbes, Viruses and Parasites in AIDS Process.

[57] Samie A., Makuwa S., Mtshali S., Potgieter N., Thekisoe O., Mbati P., Bessong P.O. (2014). Parasitic infection among HIV/AIDS patients at Bela-Bela clinic, Limpopo province, South Africa with special reference to *Cryptosporidium*. Southeast Asian J. Trop. Med. Public Health.

[58] Samra N.A., Jori F., Xiao L., Rikhotso O., Thompson P.N. (2013). Molecular characterization of *Cryptosporidium* species at the wildlife/livestock interface of the Kruger National Park, South Africa. Comp. Immunol. Microbiol. Infect. Dis..

[59] Smith G., Van den Ende J. (1986). Cryptosporidiosis among black children in hospital in South Africa. J. Infect..

[60] Steele A.D., Geyer A., Alexander J.J., Crewe-Brown H.H., Fripp P.J. (1988). Enteropathogens isolated from children with gastro-enteritis at Ga-Rankuwa Hospital, South Africa. Ann. Trop. Paediatr..

[61] Trönnberg L., Hawksworth D., Hansen A., Archer C., Stenström T.A. (2010). Household-based prevalence of helminths and parasitic protozoa in rural KwaZulu-Natal, South Africa, assessed from faecal vault sampling. Trans. R. Soc. Trop. Med. Hyg..

[62] Walters I.N., Miller N.M., Van den Ende J., Dees G.C., Taylor L.A., Taynton L.F., Bennett K.J. (1988). Outbreak of cryptosporidiosis among young children attending a day-care centre in Durban. S. Afr. Med. J..

[63] Wittenberg D.F., Smith E.G., Den Ende J.V., Becker P.J. (1987). *Cryptosporidium*-associated diarrhoea in children. Ann. Trop. Paediatr..

[64] Ng J., Yang R., McCarthy S., Gordon C., Hijjawi N., Ryan U. (2011). Molecular characterization of *Cryptosporidium* and Giardia in pre-weaned calves in Western Australia and New South Wales. Vet. Parasitol..

[65] Zambrano L.D., Levy K., Menezes N.P., Freeman M.C. (2014). Human diarrhoea infections associated with domestic animal husbandry: A systematic review and meta-analysis. Trans. R. Soc. Trop. Med. Hyg..

[66] Zhang W., Wang R., Yang F., Zhang L., Cao J., Zhang X., Ling H., Liu A., Shen Y. (2013). Distribution and genetic characterizations of *Cryptosporidium* spp. in pre-weaned dairy calves in North-eastern China’s Heilongjiang Province. PLoS ONE.

[67] Matilla F., Velleman Y., Harrison W., Nevel M. (2018). Animal influence on water, sanitation and hygiene measures for zoonosis control at the household level: A systematic literature review. PLoS Negl. Trop. Dis..

[68] Bjorkman C. (2018). Disinfection with hydrated lime may help manage cryptosporidiosis in calves. Vet. Parasitol..

[69] Grinberg A. (2002). Controlling the onset of natural cryptosporidiosis in calves with paromomycin sulphate. Vet. Rec. J..

[70] Kay D., Dufour A., Bartram J. (2012). Effectiveness of best management practices for attenuating the transport of livestock derived pathogens within catchments. Animal Waste Water Quality and Human Health.

[71] Ligda P., Claerebout E., Kostopoulou D., Zdragas A., Casaert S., Robertson L.J., Sotiraki S. (2020). *Cryptosporidium* and Giardia in surface water and drinking water: Animal sources and towards the use of a machine-learning approach as a tool for predicting contamination. Environ. Pollut..

[72] Xiao L., Bern C., Limor J., Sulaiman I., Roberts J., Checkley W., Cabrera L., Gilman R.H., Lal A.A. (2001). Identification of 5 types of *Cryptosporidium* parasites in children in Lima, Peru. J. Infect Dis..

[73] Krumkamp R., Aldrich C., Maiga-Ascofare O., Mbwana J., Rakotozandrindrainy N., Borrmann S., Caccio S.M., Rakotozandrindrainy R., Adegnika A.A., Lusingu J. (2020). Transmission of *Cryptosporidium* spp. among human and animal local contact networks in sub-Saharan Africa: A multi-country study. Clin. Infect. Dis..

[74] Daniels M.E., Shrivastava A., Smith W.A., Sahu P., Odagiri M., Misra P.R., Panigrahi P., Suar M., Clasen T., Jenkins M.W. (2015). *Cryptosporidium* and Giardia in humans, domestic animals, and village water sources in rural India. Am. J. Trop. Med. Hyg..

[75] Odeniran P.O., Ademola I.O. (2019). Epidemiology of *Cryptosporidium* infection in different hosts in Nigeria: A meta-analysis. Parasitol. Int..

[76] Raga D.K., Menkir S., Getachew Y. (2014). Prevalence of Isospora belli and *Cryptosporidium* parvum Infections among HIV Sero-positive Patients in Asella Hospital, Central Ethiopia. Res. Rev. A J. Immunol..

[77] Assefa S., Erko B., Medhin G., Assefa Z., Shimelis T. (2009). Intestinal parasitic infections in relation to HIV/AIDS status, diarrhoea and CD4 T-cell count. BMC Infect. Dis..

[78] Berahmat R., Spotin A., Ahmadpour E., Mahami-Oskouei M., Rezamand A., Aminisani N., Ghojazadeh M., Ghoyounchi R., Mikaeili-Galeh T. (2017). Human cryptosporidiosis in Iran: A systematic review and meta-analysis. Parasitol. Res..

[79] Kalantari N., Ghaffari S., Bayani M. (2018). *Cryptosporidium* spp. infection in Iranian children and immunosuppressive patients: A systematic review and meta-analysis. Casp. J. Intern. Med..

[80] Mohebali M., Yimam Y., Woreta A. (2020). *Cryptosporidium* infection among people living with HIV/AIDS in Ethiopia: A systematic review and meta-analysis. Pathog. Glob. Health.

[81] Korpe P.S., Haque R., Gilchrist C., Valencia C., Niu F., Lu M., Ma J.Z., Petri S.E., Reichman D., Kabir M. (2016). Natural history of cryptosporidiosis in a longitudinal study of slum-dwelling Bangladeshi children: Association with severe malnutrition. PLoS Negl. Trop. Dis..

[82] Kattula D., Jeyavelu N., Prabhakaran A.D., Premkumar P.S., Velusamy V., Venugopal S., Geetha J.C., Lazarus R.P., Das P., Nithyanandhan K. (2017). Natural history of cryptosporidiosis in a birth cohort in southern India. Clin. Infect. Dis..

[83] Mirzaei M. (2007). Prevalence of *Cryptosporidium* sp. infection in diarrheic and non-diarrheic humans in Iran. Korean J. Parasitol..

[84] Casemore D.P., Reeves D., Geddes A. (1988). Human cryptosporidiosis. Recent Advances in Infection.

[85] Tariuwa H.O., Ajogi I., Ejembi C.L., Awah I.J., Green P.A., Fadipe E.O., Odoba M.B. (2007). Incidence of *Cryptosporidium* infection in port-harcourt rivers state Nigeria based on regular contact with domestic animals. Niger. Vet. J..

[86] Laubach H.E., Bentley C.Z., Ginter E.L., Spalter J.S., Jensen L.A. (2004). A study of risk factors associated with the prevalence of *Cryptosporidium* in villages around Lake Atitlan, Guatemala. Brazil. J. Infect. Dis..

[87] Garvey P., McKeown P. (2009). Epidemiology of human cryptosporidiosis in Ireland, 2004-2006: Analysis of national notification data. Eurosurveillance.

[88] Siwila J. (2012). Prevalence, Characterization and Transmission of *Cryptosporidium* Species Between Animals and Humans on Dairy Farms in Zambia. Doctoral Dissertation.

[89] Erhabor O., Obunge O., Awah I. (2011). Cryptosporidiosis among HIV-infected persons in the Niger Delta of Nihgeria. Niger. J. Med..

[90] Mumtaz S., Ahmed J., Ali L. (2010). Frequency of *Cryptosporidium* infection in children under five years of age having diarrhoea in the North West of Pakistan. Afr. J. Biotechnol..

[91] Sinyangwe N.N., Siwila J., Muma J.B., Chola M., Michelo C. (2020). Factors associated with *Cryptosporidium* infection among adult HIV positive population in contact with livestock in Namwala District, Zambia. Front. Public Health.

[92] Raphael M., Mathias A., Tirah G., Mafindi M. (2017). Prevalence of *Cryptosporidium* species in HIV positive and negative patients attending Hong general hospital and Michika general hospital, Adamawa state, Nigeria. Am. J. Eng. Res..

[93] Galdas P.M., Cheater F., Marshall P. (2005). Men and health help-seeking behaviour: Literature review. J. Adv. Nurs..

[94] Noone J.H., Stephens C. (2008). Men, masculine identities, and health care utilisation. Sociol. Health Illn..

[95] Laksemi D.A., Suwanti L.T., Mufasirin M., Suastika K., Sudarmaja M. (2019). Opportunistic parasitic infections in patients with human immunodeficiency virus/acquired immunodeficiency syndrome: A review. Vet. World.

[96] Tumwine J.K., Kekitiinwa A., Bakeera-Kitaka S., Ndeezi G., Downing R., Feng X., Akiyoshi D.E., Tzipori S. (2005). Cryptosporidiosis and microsporidiosis in Ugandan children with persistent diarrhoea with and without concurrent infection with the human immunodeficiency virus. Am. J. Trop. Med. Hyg..

[97] Karshima S.N., Karshima M.N. (2020). Epidemiology of *Cryptosporidium* Infections among People Living with HIV/AIDS in Nigeria: Results of Systematic Review and Meta-analysis. Acta Parasitol..

[98] Wang R.J., Li J.Q., Chen Y.C., Zhang L.X., Xiao L.H. (2018). Widespread occurrence of *Cryptosporidium* infections in patients with HIV/AIDS: Epidemiology, clinical feature, diagnosis, and therapy. Acta Trop..

[99] Nsagha D.S., Njunda A.L., Assob N.J.C., Ayima C.W., Tanue E.A., Kwenti T.E. (2015). Intestinal parasitic infections in relation to CD4+ T cell counts and diarrhoea in HIV/AIDS patients with or without antiretroviral therapy in Cameroon. BMC Infect. Dis..

[100] Khurana S., Chaudhary P. (2018). Laboratory diagnosis of cryptosporidiosis. Trop. Parasitol..

